# Proteolysis of Gingival Keratinocyte Cell Surface Proteins by Gingipains Secreted From *Porphyromonas gingivalis* – Proteomic Insights Into Mechanisms Behind Tissue Damage in the Diseased Gingiva

**DOI:** 10.3389/fmicb.2020.00722

**Published:** 2020-04-28

**Authors:** Katarina Hočevar, Matej Vizovišek, Alicia Wong, Joanna Kozieł, Marko Fonović, Barbara Potempa, Richard J. Lamont, Jan Potempa, Boris Turk

**Affiliations:** ^1^Department of Biochemistry, Molecular and Structural Biology, Jožef Stefan Institute, Ljubljana, Slovenia; ^2^International Postgraduate School Jožef Stefan, Ljubljana, Slovenia; ^3^Department of Microbiology, Faculty of Biochemistry, Biophysics and Biotechnology, Jagiellonian University, Kraków, Poland; ^4^Department of Oral Immunology and Infectious Diseases, University of Louisville School of Dentistry, Louisville, KY, United States; ^5^Faculty of Chemistry and Chemical Technology, University of Ljubljana, Ljubljana, Slovenia

**Keywords:** gingipains, proteases of *Porphyromonas gingivalis*, cell surface proteolysis, shedding, anoikis, proteomics

## Abstract

*Porphyromonas gingivalis*, the main etiologic agent of periodontitis, secretes cysteine proteases named gingipains. HRgpA and RgpB gingipains have Arg-specificity, while Kgp gingipain is Lys-specific. Together they can cleave an array of proteins and importantly contribute to the development of periodontitis. In this study we focused on gingipain-exerted proteolysis at the cell surface of human gingival epithelial cells [telomerase immortalized gingival keratinocytes (TIGK)] in order to better understand the molecular mechanisms behind tissue destruction in periodontitis. Using mass spectrometry, we investigated the whole sheddome/degradome of TIGK cell surface proteins by *P. gingivalis* strains differing in gingipain expression and by purified gingipains, and performed the first global proteomic analysis of gignpain proteolysis at the membrane. Incubation of TIGK cells with *P. gingivalis* resulted in massive degradation of proteins already at low multiplicity of infection, whereas incubating cells with purified gingipains resulted in more discrete patterns, indicative of a combination of complete degradation and shedding of membrane proteins. Most of the identified gingipain substrates were molecules involved in adhesion, suggesting that gingipains may cause tissue damage through cleavage of cell contacts, resulting in cell detachment and rounding, and consequently leading to anoikis. However, HRgpA and RgpB gingipains differ in their mechanism of action. While RgpB rapidly degraded the proteins, HRgpA exhibited a much slower proteolysis indicative of ectodomain shedding, as demonstrated for the transferrin receptor protein 1 (TFRC). These results reveal a molecular underpinning to *P. gingivalis*-induced tissue destruction and enhance our knowledge of the role of *P. gingivalis* proteases in the pathobiology of periodontitis. Proteomics data are available via ProteomeXchange with identifier PXD015679.

## Introduction

*Porphyromonas gingivalis* is a gram-negative anaerobe, and the main causative agent of a chronic oral inflammatory disease – periodontitis (Lamont and Jenkinson, 1998). The proliferation of *P. gingivalis* and other oral pathogens leads to severely inflamed and bleeding gums, deepening of the periodontal pocket, gingival tissue destruction and, in the most advanced stages, alveolar bone destruction and tooth loss. Cysteine proteases secreted by *P. gingivalis*, known as gingipains, are believed to be the most potent virulence factors of the organism (Holt et al., 1999; Fitzpatrick et al., 2009; Lopes et al., 2012; Hajishengallis, 2015). The gingipain family is composed of three related proteases with strict Arg- or Lys-specificity. Gingipains RgpA and RgpB (also termed R-gingipains) cleave their substrates exclusively at the Arg-Xaa bond, while gingipain Kgp (also known as K-gingipain) hydrolyzes its targets solely at Lys-Xaa peptide bonds (Chen et al., 1992; Pike et al., 1994; Curtis et al., 2000). The main difference between RgpA and RgpB is that the former is secreted as a large, non-covalent complex, consisting of the catalytic and hemagglutinin/adhesion (HA) domains. The complex of the RgpA catalytic domain and HA domains is termed HRgpA and has molecular weight of ∼95 kDa (Curtis et al., 2000; Imamura et al., 2000). On the other hand, RgpB is a single chain protein with amino-acid sequence almost identical to that of the RgpA catalytic domain (Potempa et al., 1998; Imamura et al., 2000). Kgp also contains HA domains that are capable of interacting with the related adhesion domains of HRgpA and thus both gingipains can form a very potent proteolytic HRgpA-Kgp complex with two active sites and specificity for both Arg-Xaa and Lys-Xaa peptide bonds (Bhogal et al., 1997; Takii et al., 2005). With their specific and very potent proteolytic activities, gingipains are important for bacterial survival at inflamed sites of periodontal pockets, and for the pathological outcome of the disease. In particular, gingipains contribute to the ability of *P. gingivalis* to adhere to other bacteria and oral surfaces, are responsible for acquisition of nutrients essential for bacterial growth, and cause immune evasion and subversion (reviewed in Kuramitsu, 1998; O’Brien-Simpson et al., 2003; Fitzpatrick et al., 2009; Guo et al., 2010). Furthermore, gingipains can degrade specific host cell-surface proteins, which can result in imbalanced signaling, cell detachment and anoikis, a form of cell death due to loss of intercellular connections (reviewed in Kuramitsu, 1998; Chiarugi and Giannoni, 2008; Fitzpatrick et al., 2009; Guo et al., 2010).

The ability of gingipains to cleave host cell surface proteins and release their soluble domains led to the proposal that gingipains may act as sheddases (Hocevar et al., 2018). Generally, all sheddases release entire ectodomains of membrane-anchored proteins by proteolytic cleavage in the proximity of the membrane [up to 20 amino acid residues away from the membrane (Lichtenthaler et al., 2018)]. Once released into the extracellular milieu, soluble ectodomains often exert new biological functions (Arribas and Borroto, 2002). However, the demarcation between shedding and complete degradation is very narrow, as it was shown that shedding is often followed by degradation of ectodomains as periodontal disease progresses (Hocevar et al., 2018). Several proteins were thus found to be shed by gingipains, including EMMPRIN (Feldman et al., 2011), Syndecan-1 (Andrian et al., 2006), CD46 (Mahtout et al., 2009), TREM-1 (Bostanci et al., 2013; Belibasakis et al., 2014), and CD14 (Sugawara et al., 2000). However, these studies were conducted using different cellular models, purified gingipains, *P. gingivalis* or even multi species biofilms, making a comparison difficult.

Knowledge of the host cell substrates which are preferentially cleaved by gingipains will enhance our understanding of the complexity of *P. gingivalis*-mediated tissue destruction. Therefore, our aim was to investigate gingipain-derived proteolysis of the cell surface proteins on gingival keratinocytes (TIGK). Our first questions was, whether proteolysis is actually shedding or degradation of the mentioned proteins. Furthermore, we wanted to understand which proteins get proteolysed and how this could effect the TIGK cells or how bacteria benefit from it. To address these questions, we used mass spectrometry to investigate the whole sheddome/degradome elicited by different *P. gingivalis’* gingipains from the surface of TIGK cells. The identified membrane targets were predominantly adhesion molecules, suggesting that gingipains cause tissue destruction through elimination of cell contacts and consequent induction of anoikis. Moreover, the results suggest that degradation of extracellular proteins by gingipains is likely the main mode of action of these important bacterial enzymes.

## Materials and Methods

### Gingipains, Inhibitors, and Buffers

Gingipains were purified from *P. gingivalis* culture supernatant as described earlier (Potempa and Nguyen, 2008). Buffers TNC (100 mM Tris, pH 7.5, 150 mM NaCl, 5 mM CaCl_2_, 10 mM L-Cys) and TNC with added 0.05% Tween-20 (TNCT) were applied for optimal gingipain activity. TNC was used for gingipain or *P. gingivalis* treatment of the TIGK cell line, while TNCT was used for *in vitro* tests and active site titration. Specific R-gingipain inhibitor KYT-1 and specific K-gingipain inhibitor KYT-36 were purchased from Peptide Institute (#4395-v and #4396-v, respectively). Active site titration was performed to determine the concentration of active gingipains. Gingipains were titrated using KYT-1 and KYT-36, with L-BApNA (Bachem, #4000792) and Ac-Lys-pNA (Bachem, #4004444) as substrates for R-gingipains and K-gingipain, respectively. Gingipains were diluted in TNCT and incubated at 37°C for 15 min. In transparent 96-wells 50 μL of gingipain at final concentration of 10 nM and 50 μL of appropriately diluted inhibitor were mixed to yield final concentrations of 0, 0.1, 0.2, 0.3, 0.4, 0.6, 1, 1.5, 2, 2.5, 3.0, 4.0, 6.0, and 8.0 nM. After 15-min incubation 100 μL of the substrate were added to the final concentration of 200 μM. Absorbance of the released product was then continuously measured using Infinite M1000 Pro (Tecan) microplate reader at a wavelength of 405 nm and at 37°C. Relative velocities of substrate cleavage were plotted against inhibitor concentrations, and active gingipain concentration was calculated with linear regression analysis.

### Cell Culture

TIGK cells (Moffatt-Jauregui et al., 2013) were grown to confluence in growth media with supplements (KGM Bullet Kit; Lonza). Cells were cultivated at constant temperature (37°C) and controlled atmosphere (5% CO_2_, saturated air).

### Bacterial Strains and Cultivation

*P. gingivalis* strains used in this study were wild-type W83 (American Type Culture Collection, Rockville, MD, United States) and its isogenic single, double and triple protease-null mutants, Δ*kgp* (deletion of the K-gingipain encoding gene), Δ*rgpAB* (deletion of both R-gingipain encoding genes) and Δ*kgp*Δ*rgpAB* (deletion of all three gingipain-encoding genes). The general procedure for construction of the mutants has been described elsewhere (Shi et al., 1999). Bacteria were maintained on blood agar and cultivated in Bulion Schädler broth at 37°C in an anaerobic chamber (90% N_2_, 5% CO_2_, 5% H_2_).

### Treatment of TIGK Cells With *P. gingivalis*

*P. gingivalis* is sufficient cells in the late exponential/early stationary phase were collected by centrifugation for 5 min at 5,000 × *g*, 4°C. The bacterial pellet was washed twice with TNC (5 min at 5,000 × *g*, 4°C) and bacterial cells were resuspended in TNC buffer at absorbance A_600 *nm*_ = 1, which is equivalent to 10^9^ bacterial cells/mL. Suspended bacteria were added to the adherent TIGK cells, which were previously washed with DPBS twice. We used confluent TIGK cells, grown on 15-cm plates. In each experiment 36 million cells per sample were used. TIGK cells were then infected with *P. gingivalis* in culture at MOI (multiplicity of infection) 25, 100, or 500. After 45 min incubation, gingipain activity was blocked with addition of inhibitors (1 μM final concentration of each inhibitor, KYT-1 and KYT-36). Supernatant was collected, centrifuged at 300 × *g* for 5 min to remove remaining TIGK cells, before centrifugation (5 min at 5,000 × *g*) to remove bacterial cells. Supernatant was then analyzed by SDS-PAGE or Western blotting.

### Treatment of TIGK Cells With Purified Gingipains

Active site-titrated gingipains (Supplementary Figure 1) were diluted in TNC buffer and incubated at 37°C for 15 min in order to be fully activated. Adherent confluent TIGK cells (36 millions of cells per sample) were washed with DPBS twice, then treated with buffer containing gingipains at final active concentrations of 4, 40, 75, or 100 nM for 45 min at 37°C. Conditioned media were collected in centrifugal tubes and gingipain inhibitors (at concentration 1 μM of each inhibitor; see above) were added to the solution before the samples were centrifuged twice at 300 × *g* and then at 3,000 × *g* for 5 min in order to remove cells and cell debris, respectively. Next, samples were concentrated to a final volume of 200 μL using membrane concentrators (MWCO 3000, Millipore). As a negative control, gingipains inhibited with KYTs were used. Briefly, KYTs (200 μM final concentration) were added to activated gingipain solutions and incubated for 15 min at 37°C prior to treatment of TIGK cells (see above). Samples were then used either for mass spectrometry analysis or for SDS-PAGE/Western blotting.

In the microscopy experiments, TIGK cells were cultivated in 6-well plates until confluency. Gingipains were applied at active concentrations of 4, 40, 100, and 250 nM and photographs taken using a light microscope (Olympus IX 81) with 10x zoom, phase contrast and a Hamamatsu Orca R2 camera every 5 min for 1 h.

### Mass Spectrometry Sample Preparation

Samples were prepared for mass spectrometry analysis by the “in-gel” protocol (Vizovisek et al., 2015), where they were separated on 12.5% SDS-PAGE precast Tris-Gly gels (Lonza). The gels were stained with either Coomassie Brilliant Blue or Silver Stain. Each protein lane was cut into six bands that were further cut into 1 mm^3^ pieces (referred to as samples in the following text). Samples were destained [25 mM NH_4_HCO_3_ (Fluka Biochemica) in 50% acetonitrile (JT Baker)], washed with acetonitrile and vacuum dried, before rehydrated in reducing solution (10 mM DTT (Fluka Biochemica) in 25 mM NH_4_HCO_3_) and incubated at 56°C for 45 min. Next, samples were soaked in the alkylating solution [55 mM iodoacetamide (Amersham Biosciences) in 25 mM NH_4_HCO_3_] and incubated for 30 min in the dark. Subsequently, the samples were first washed with 25 mM NH_4_HCO_3_, followed by an acetonitrile washing step and finally vacuum dried before trypsinization. Next, samples were rehydrated in trypsinization buffer (Promega) with sequencing-grade modified porcine trypsin (Promega) and left to be digested overnight at 37°C. Next day, the digest was collected and the remaining peptides were extracted from the gels using the extraction solution [50% acetonitrile, 5% formic acid (Fluka)]. The samples were then concentrated on a speedvac and loaded on C18 tips for desalting. The C18 tips were prepared by packing Empore C18 disks (Sigma Aldrich) in 200 μL pipet tips as described (Rappsilber et al., 2007). Briefly, the tips were conditioned with 50 μL 100% methanol (Merck), followed by 80% acetonitrile containing 3% acetic acid (VWR Chemicals) and equilibrated with 0.1% formic acid (JT Baker). Samples were loaded on the tips and washed twice with 0.1% formic acid (JT Baker). The samples were eluted with 50% acetonitrile containing 0.1% formic acid (JT Baker) and concentrated to 10 μL before proceeding with the MS analysis. Experiments for mass spectrometry were repeated at least three times.

### Proteomic Identification of Extracellular Gingipain Substrates

#### LC-MS/MS

LC-MS/MS analysis was performed on an EASY-nanoLC II HPLC (Thermo Fischer Scientific) in-line with LTQ Orbitrap Velos mass spectrometer (Thermo Fischer Scientific). The peptide samples were loaded on a C18 trapping column (EASY-Column^TM^, Thermo Fischer Scientific) and separated on a C18 PicoFrit^TM^ AQUASIL analytical column (New Objective) with a 90 min linear gradient (5–40% solvent B, 0.1% formic acid in acetonitrile) at a constant flow rate of 300 nL/min. The LC-MS/MS was operated via Xcalibur software (Thermo Fischer Scientific) and MS spectra were acquired in the Orbitrap analyzer at 30,000 resolution and a mass range of 300–2000 m/z. HCD fragmentation of the nine most intense precursor ions from the full MS was used to generate MS/MS spectra which were recorded at resolution 7,500. Only precursors with a charge state ≥ 2 were selected for further fragmentation.

Database searches were performed using the MaxQuant version 1.5.6.0 (Cox and Mann, 2008; Cox et al., 2011) against the UniProt-derived human reference proteome (UniProtKB, *Homo sapiens*, canonical database containing 20,316 entries). The database searches of the peptide spectra were performed using the standard trypsin specificity rule KR/-P allowing for one missed cleavage. The following modifications were set in the searches: carbamidomethylation of cysteine residues (+57.0215 Da) as fixed modification, oxidation of methionine (+15.9949 Da) and acetylation of protein N-termini (+42.0106 Da) as variable modifications. The mass tolerances of the precursor ion and fragment ion were 4.5 ppm and 0.5 Da and 1% FDR settings were used for protein identification. MaxQuant was used for the label-free quantification using default settings. For relative quantification, spectral counting of razor and unique peptides was used as described previously (Liu et al., 2004). The mass spectrometry data have been deposited to the ProteomeXchange Consortium via the PRIDE (Perez-Riverol et al., 2018) partner repository with the dataset identifier PXD015679.

#### Data Processing and Identification of Potential Targets

The data were processed with Perseus to filter potential contaminants, reverse sequences and single peptide identifications. To minimize the number of false positives only proteins with more than two counts per protein were considered in the analysis (Sobotic et al., 2015). Protein SCR (spectral count ratios) were calculated by dividing spectral counts in gingipain-treated samples by spectral counts in the negative control (samples treated with inhibited gingipains). Spectral counts of all proteins were increased by 1 to avoid division by zero. SCR values between 0.33 and 3 (0.33 < SCR < 3) abundance of selected protein were considered non-changed between negative control and the sample. SCR lower than 0.33 (SCR ≤ 0.33) was considered a decrease of protein abundance in the sample compared to the negative control, while SCR greater than 3 (SCR ≥ 3) was considered an increase in protein abundance in the sample. For the potential target list, we included only extracellular/membrane proteins that had a SCR ratio ≥ 3.

### Structural and Functional Analysis of Potential Substrates

Potential extracellular substrates of gingipains were analyzed according to their structure, molecular mass and biological function. Gene ontology was performed using g:Profiler (Reimand et al., 2007, 2016) web interface, while structural analysis was done with the InterPro interface (Mitchell et al., 2014) and with SMART.embl-heidelberg.de.

### Western Blotting

Samples of treated TIGK cells were loaded on a 12% Tris-Glycine gel for SDS-PAGE (Lonza) in equal volumes. Western Blot and immune-detection used polyclonal goat anti-TFRC (Transferrin receptor protein-1) antibodies (R&D, #af2474) at dilution 1:500 and polyclonal donkey anti-goat secondary antibodies (Abcam, #Ab6885) at dilution 1:3000. Detection of bands was performed using ECL reagent (Amersham Pharmacia Biotech).

## Results

**FIGURE 1:**
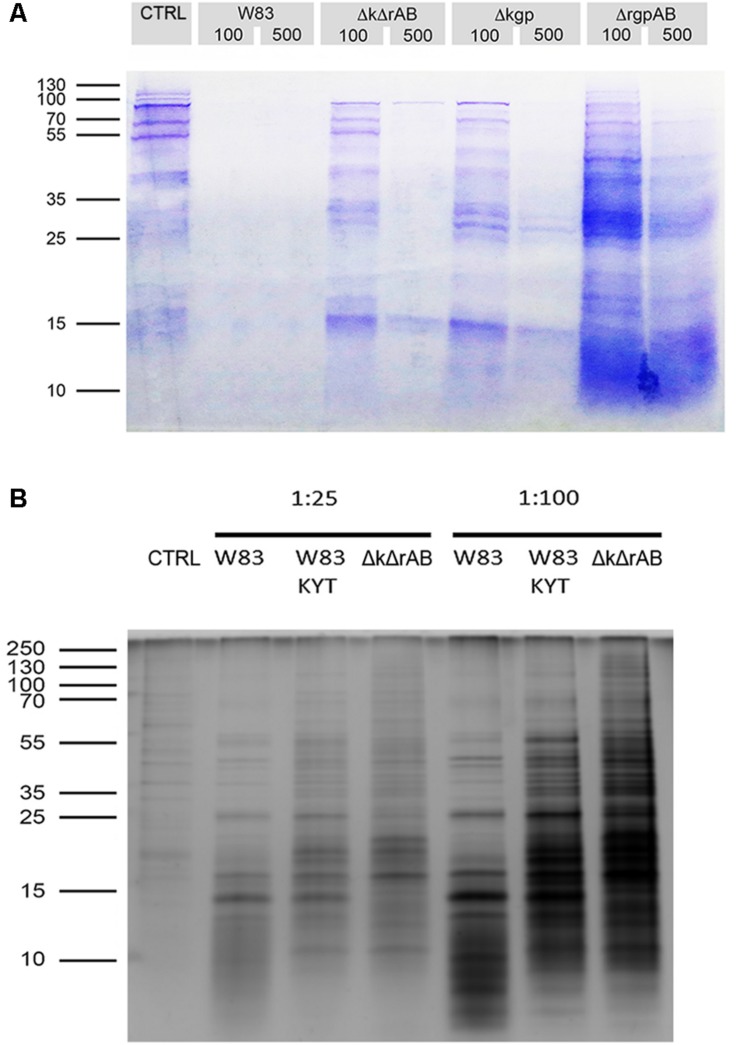
Treatment of TIGKs with *Porphyromonas gingivalis* results in degradation of cell surface proteins. **(A)** SDS-PAGE gel stained with Coomassie Blue showing the amounts of proteins in samples obtained by treating TIGKs with different strains of *P. gingivalis*, including W83 – wild type, Δk*gpΔrgpAB* – knock-out of all gingipains (marked Δk*ΔrAB* in the figure), Δk*gp* – knock-out of Kgp and *ΔrgpAB* – inactivation of RgpA and RgpB. Mostly blank lanes indicate extensive protein degradation. Intact TIGK cells were treated with *P. gingivalis* strains at MOI 100 and MOI 500, while TNC buffer was used as a control. Equal volumes of all samples (20 μL) were loaded on the gel. **(B)** Silver stained SDS-PAGE gel of sheddomes, obtained by treating intact TIGKs with different numbers of W83 and Δk*gpΔrgpAB* (marked Δk*ΔrAB* in the figure) bacterial cells (MOI 25 and MOI 100). W83 was used with and without treatment with gingipain inhibitors – a mixture of KYT-1 and KYT-36, to compare the effect of the presence of inhibited gingipains and the total absence of gingipains in Δk*gpΔrgpAB*. TIGKs treated with TNC buffer alone constituted the negative control (CTRL).

To identify extracellular substrates of gingipains, we first performed a comparative evaluation of proteolysis of gingipain substrates on the surface of TIGK cells, using *P. gingivalis* strains expressing: all gingipains (strain W83), RgpAB (strain Δ*kgp)*, Kgp (strain Δ*rgpAB*), or no gingipains (strain Δ*kgp*Δ*rgpAB*). Prior to each experiment we checked and confirmed cell and bacterial viability (Supplementary Figure 2). After the treatment of TIGK cells with *P. gingivalis* strains at two bacteria to cell ratios (MOI 100 and MOI 500), conditioned media were collected and analyzed by SDS-PAGE using a Coomassie Blue stain which showed that the concentration of proteins in the samples was very low at MOI 500 (Figure 1A) indicative of massive protein degradation. However, at MOI 100 the protein concentration in samples was higher and we selected these for further analysis. Moreover, we noticed differences between the amounts of proteins in the supernatants obtained with different strains of *P. gingivalis*. In the case of W83, there were very low amounts of proteins on the gel, while strains Δk*gp*, Δ*rgpAB*, and Δ*kgp*Δ*rgpAB* yielded increased amounts of proteins with Δ*rgpAB* showing the highest yield. This suggests substantial degradation of the outer membrane proteins by *P. gingivalis* gingipains. However, it should be noted that TIGK cells even in the absence of bacteria secrete some proteins.

In order to further prevent degradation as observed at MOI 100, we lowered the number of bacteria to MOI 25. SDS-PAGE with silver staining at MOI 25 revealed substantially lower amounts of small protein fragments on the gel, which suggests diminished proteolytic degradation (Figure 1B). Furthermore, corroboration that the observed degradation is a direct consequence of gingipain activity was provided by the addition of specific gingipain inhibitors (KYTs) to the sample. With the W83 strain this resulted in a significant shift toward higher MW proteins on the gel, which was even more pronounced when the gingipain-null strain was used. However, just the presence of bacteria was found to elicit enhanced secretion or degradation of proteins from TIGK cells, which could be a consequence of other *P. gingivalis* effectors, such as fimbriae, LPS and secretion of other proteases.

**FIGURE 2:**
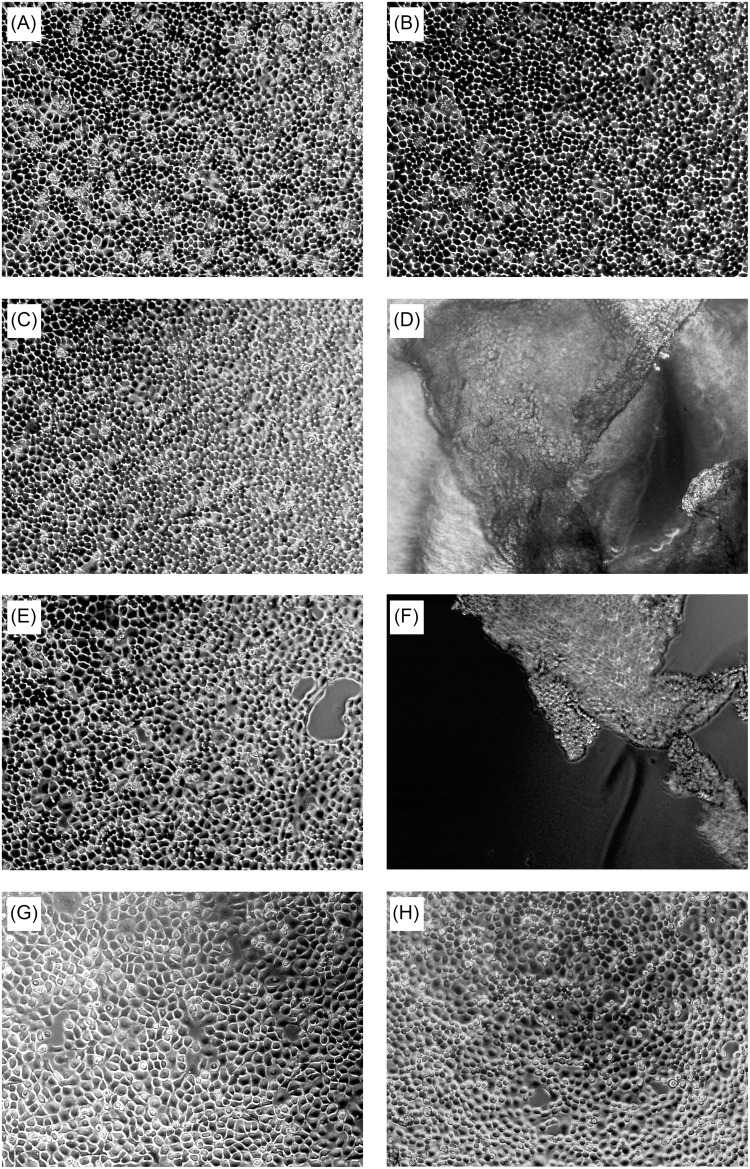
Concentration-dependent effect of gingipains on TIGKs after 45-min treatment. Intact TIGKs were treated with TNC buffer as a control or with gingipains at different concentrations to compare the effect of bacterial proteases on the confluent TIGKs. The pictures were taken using light microscopy. The cell morphology of TIGKs treated with gingipains without incubation (*t* = 0 min) **(A)**, after 45 min of incubation with: TNC buffer only **(B)**, 4 nM RgpB **(C)**, 40 nM RgpB **(D)**, 4 nM HRgpA **(E)**, 40 nM HRgpA **(F)**, 4 nM Kgp **(G)**, and 40 nM Kgp **(H).**

In order to avoid these effects, we treated cells separately with each of the purified gingipains (Kgp, HRgpA, or RgpB). In the first experiment, we examined the effect of these gingipains at two different concentrations (4 and 40 nM) on TIGK cells using light microscopy, and followed changes in cell morphology over time (Figure 2). At the lower concentration of gingipains (4 nM), TIGK cells were visually identical to untreated cells. In contrast, a 45-min treatment with RgpB and HRgpA at the 40 nM concentration resulted in cell rounding and detachment; although the cells were still viable, as judged by ReadyProbes Cell Viability Imaging Kit (Supplementary Figure 3). Similar observations have been made for fibroblast cell cultures treated with gingipains (Baba et al., 2001). Conversely, Kgp at the same concentration and incubation time did not detach cells. Therefore, we used a higher concentration of Kgp (100 nM) to yield approximately the same numbers of detached cells after 30 min incubation as was obtained with Rgp treatment (Supplementary Figure 4). This result is in agreement with results from the other experiments with gingipains, reporting that Kgp is less potent compared to Rgps (Tada et al., 2003).

**FIGURE 3:**
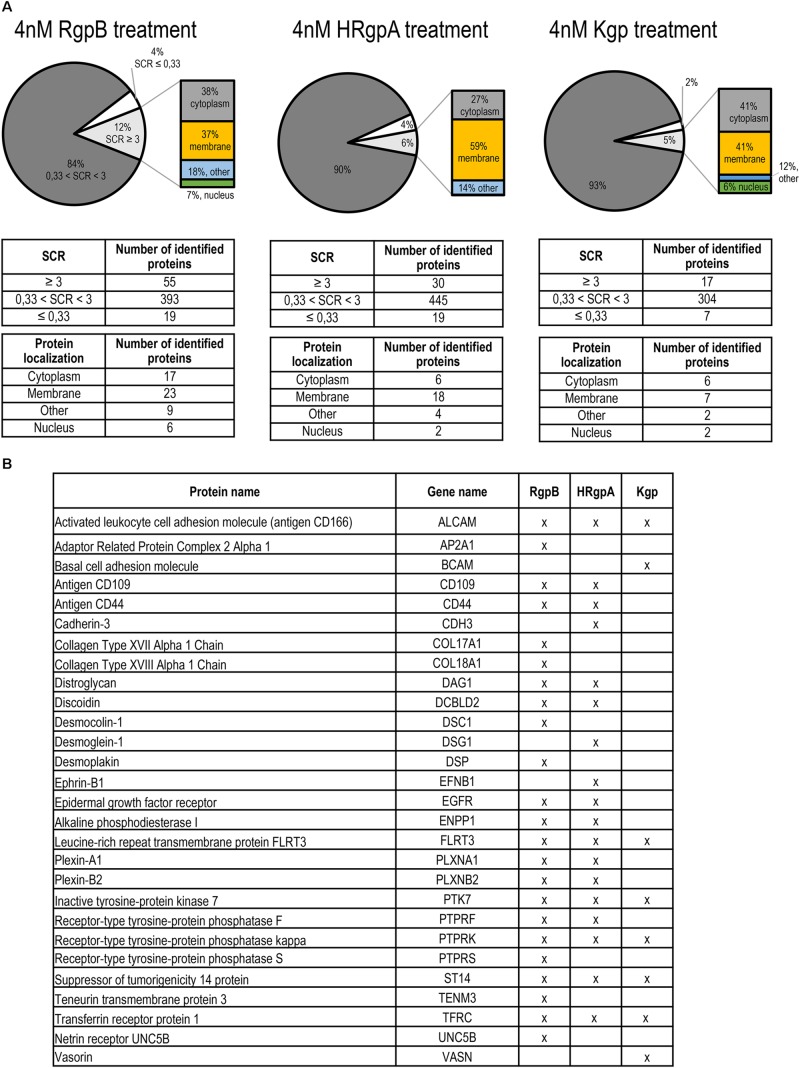
Proteomic identification of gingipain substrates on the TIGKs cell surface. **(A)** Distribution by SCR and cell localization of proteins in samples identified by mass spectrometry. Intact TIGKs were treated with purified gingipains RgpB, HRgpA, and Kgp. The pie charts for the RgpB, HRgpA, and Kgp treatment show the ratios between the proteins with decreased abundance (SCR ≤ 0.33), unchanged abundance (0.33 < SCR < 3) and increased abundance (SCR ≥ 3) after gingipain treatment. Proteins with SCR ≥ 3 were further divided into four categories, depending on their cellular localization (membrane, cytoplasm, nucleus, and other locations). **(B)** List of potential gingipain targets detected in individual gingipain sheddomes. Only proteins localized on the cell membrane, identified with a minimum two peptides and with a minimum three-fold increase in SCR were considered.

Based on these results, we selected a 45-min treatment of TIGK cells with gingipains at 37°C for further experiments, which is sufficient for proteolysis without killing the cells. We used two gingipain concentrations (4 and 75 nM) to model early and advanced stages of periodontitis. Both concentrations are physiologically relevant as the measured concentration of gingipains at inflamed sites using different methods were mostly at low nanomolar concentrations, while the highest measured concentration was 1.5 μM (Popadiak et al., 2007; Guentsch et al., 2011; Wilensky et al., 2014). In addition, the 75 nM gingipain concentration allows also for evaluation of Kgp proteolysis, which is very low at 4 nM concentration. Following TIGK cell treatment, mass spectrometry with spectral counting (Li et al., 2012) was used to measure the differences in protein abundances in the supernatants with Spectral count ratio (SCR) representing relative difference in protein abundance between protease treated sample and negative control. The largest group of proteins identified in the samples were proteins with an unchanged abundance (Figure 3A and Supplementary Figure 5A). Comparison of proteomic data between lower (4 nM) and higher (75 nM) concentrations of gingipains showed that in the latter case the number of proteins with reduced abundance was significantly higher for HRgpA and Kgp (Supplementary Figure 5) at 75 nM compared to the lower concentration where the number of identified proteins with SCR ≤ 0.33 represented a relatively small percentage of hits. With only seven identified proteins exhibiting SCR ≤ 0.33, Kgp also demonstrated the lowest protein degradation rate. In contrast, the numbers of identified proteins for RgpB treatment were similar at the two concentrations, although the total number of proteins identified was also higher at 75 nM concentration.

**FIGURE 4:**
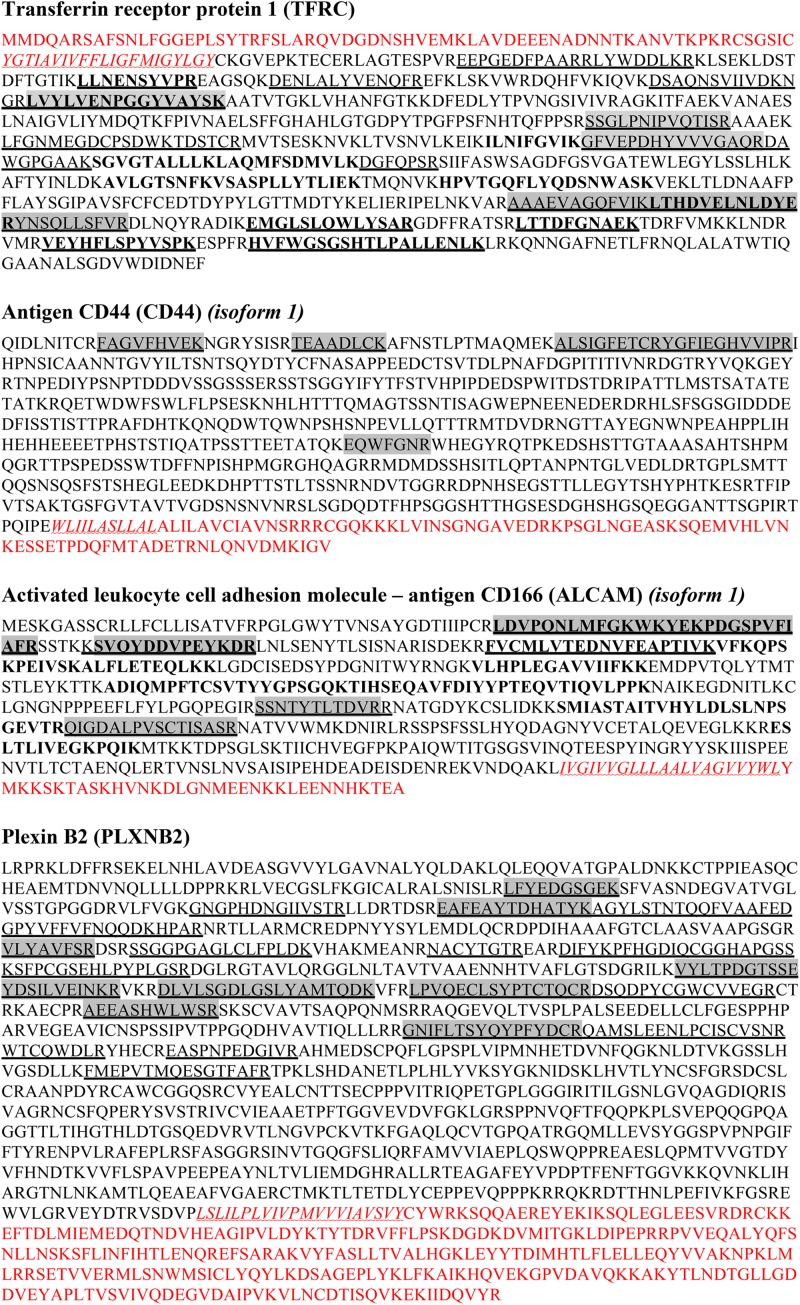
Amino acid sequences of identified membrane proteins based on tryptic peptides. The amino acid sequences of some identified membrane/extracellular proteins are shown. For each protein, multiple peptides were identified. They were all located solely in the extracellular region and none of the identified peptides was located in transmembrane or cytosolic domains. LEGEND: Red are cytosolic regions of the sequence, *red wave underlined and in italics* are transmembrane regions, extracellular domains are marked in black. Underlined are peptides obtained with RgpB treatment of TIGK cells, shaded in gray are peptides cleaved by HRgpA, **in bold** are peptides cleaved by Kgp.

To investigate gingipain-mediated shedding we therefore focused on the proteomic data obtained by treating TIGK cells with lower concentrations of gingipains, where less degradation was observed. The majority of proteins with increased abundance were found to be membrane proteins, closely followed by cytoplasmic proteins. Indeed, more than one third of all proteins were membrane proteins, consistent with proteolysis occurring on the surface of intact keratinocytes. We identified 28 putative membrane substrates of gingipains (Figure 3B), the majority of which were detected in the samples with RgpB, which has proven to be the most potent protease, followed by HRgpA and Kgp with the lowest number of identified membrane proteins. An analysis of the identified tryptic peptides in the membrane substrates showed that they were all found on the extracellular side, in agreement with their shedding from the membrane as shown for Transferrin receptor protein-1 (TFRC), CD44, Activated leukocyte cell adhesion molecule (ALCAM, CD166) and Plexin B2 (PLXNB2) (Figure 4). RgpB generated the largest number of peptides, consistent with the finding that it was the most potent protease. Moreover, a vast number of peptides generated by RgpB and HRgpA were identical, consistent with their highly similar specificities. In addition, there were even a number of shared peptides between Kgp and the two Arg-specific proteases, which could, however, be attributed to the fact that the peptides for mass spectrometric analyses were generated by trypsin that has a mixed Arg/Lys specificity and could thereby trim the peptides. Because of the smaller size of the tryptic peptides, the exact cleavage site(s) could not have been identified. However, at least in the case of TFRC one can speculate that RgpB and HRgpA cleaved the bond between Arg109-Glu110 or even between Arg100-Leu101, which is only 12 residues from the membrane. On the other hand, Kgp was cleaving a little bit downstream between Lys145-Leu146, or eventually after Lys130 or Lys134, depending on the trypsin cleavage. The identified peptides for the other proteins were further away from the membrane so it is difficult to conclude whether there was more shedding or degradation.

**FIGURE 5:**
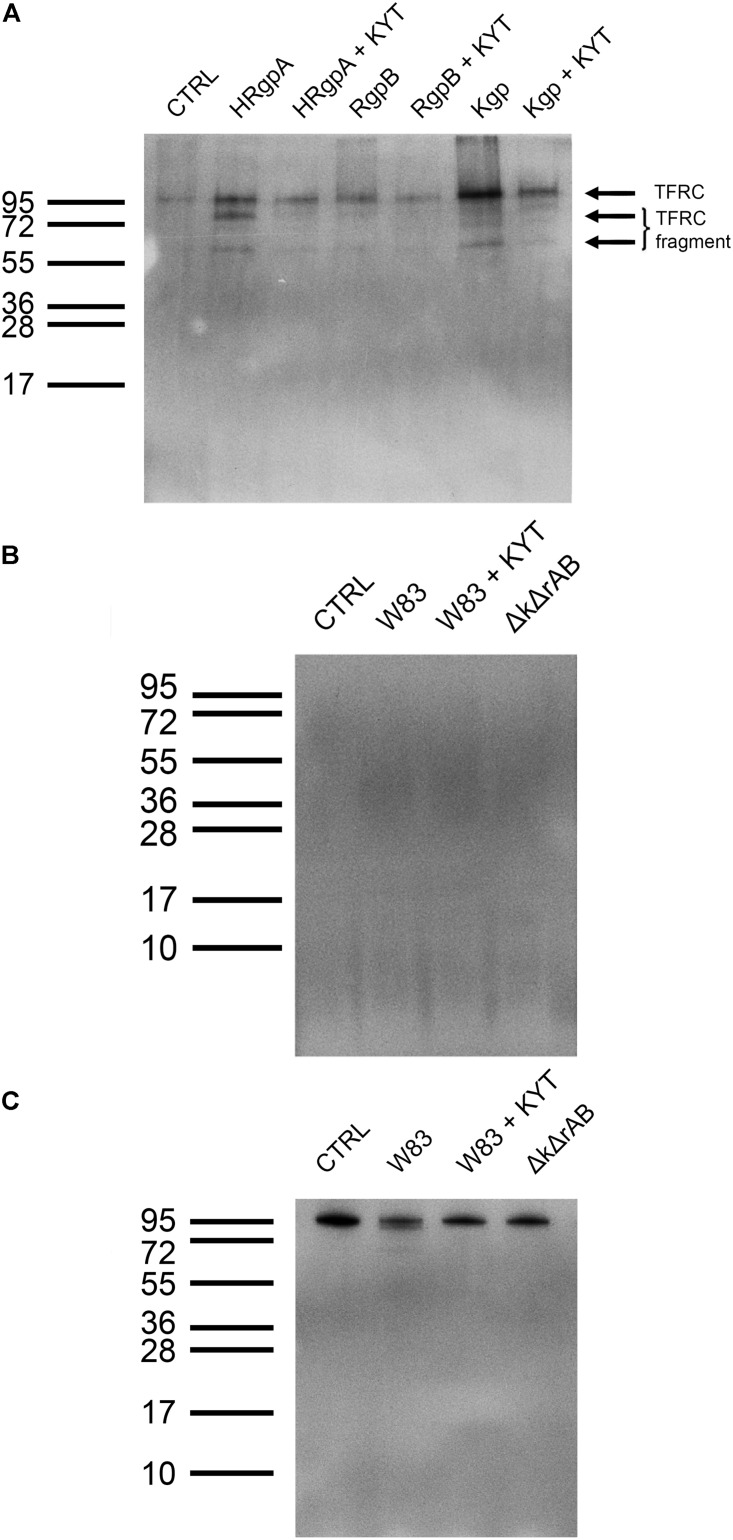
Validation of shedding target Transferrin receptor protein-1. **(A)** Immunoblot analysis of Transferrin receptor protein-1 in the sheddome of TIGK cells treated with 4 nM HRgpA, RgpB, and Kgp for 45 min using anti-TFRC antibodies reacting with the 95 kDa form of TFRC. ECL reagent and chemiluminescence were used for detection of the bands. Exposure time was 5 min. **(B)** Immunoblot of sheddomes of TIGKs treated with *P. gingivalis* (W83, W83 with added inhibitors, KYT and Δk*gpΔrgpAB*, marked as Δk*ΔrAB* in the figure) at MOI 100 for 45 min. Exposure time: 5 min. **(C)** Immunoblot of TIGK cell lysates after infection with *P. gingivalis* (W83, W83 with added inhibitors, KYT and Δk*gpΔrgpAB*, marked as Δk*ΔrAB* in the figure) at MOI 100 for 45 min. Exposure time was 5 min.

Because of that and because of the high number of identified peptides, we focused on TFRC for further validation. Using immunological detection by Western Blot (Figure 5A) we detected TFRC in the sheddome of TIGK cells treated with 4 nM Kgp, which confirmed our mass spectrometry results (Figure 3B). Interestingly, TIGK cells treated with R-gingipains demonstrated a clear difference in the efficiency in TFRC shedding by HRgpA and RgpB. Treatment with RgpB resulted in only a slight increase in the TFRC band intensity on the immunoblot, suggesting instant degradation of the solubilized receptor. Conversely, in conditioned medium of TIGK cells incubated with HRgpA, the shed receptor was clearly visible along with the degradation products (Figure 5A). The larger cleaved product (75 kDa) correspond to the receptor shortened for around 140 residues, which is essentially the whole extracellular part, whereas the 60 kDa product seen with all gingipains is a further degradation product. Again, these results corroborated the mass spectrometry analysis, which detected TFRC-derived peptides in the conditioned medium of gingipain-R treated TIGK cells.

Western Blotting of the sheddomes from TIGK cells treated with *P. gingivalis* did not reveal any bands, indicating the absence of the Transferrin receptor protein-1 in the sheddomes (Figure 5B). This could be attributed to complete degradation of the Transferrin receptor. However, as none of the samples showed any bands (including samples with added gingipain inhibitors, W83 + KYT, and the gingipain-null Δ*kgp*Δ*rgpAB*) we speculate that there are other proteases, not just gingipains, involved in TFRC degradation. Furthermore, we noticed a decrease in the thickness of the band for TFRC in the lysate of TIGK cells infected with W83 (Figure 5C). This is further confirmation that gingipains and, possibly, other proteases do shed and degrade this receptor. However, in some cases the sheddome protein material is at very low concentration, thereby precluding detection by Western Blotting.

**FIGURE 6:**
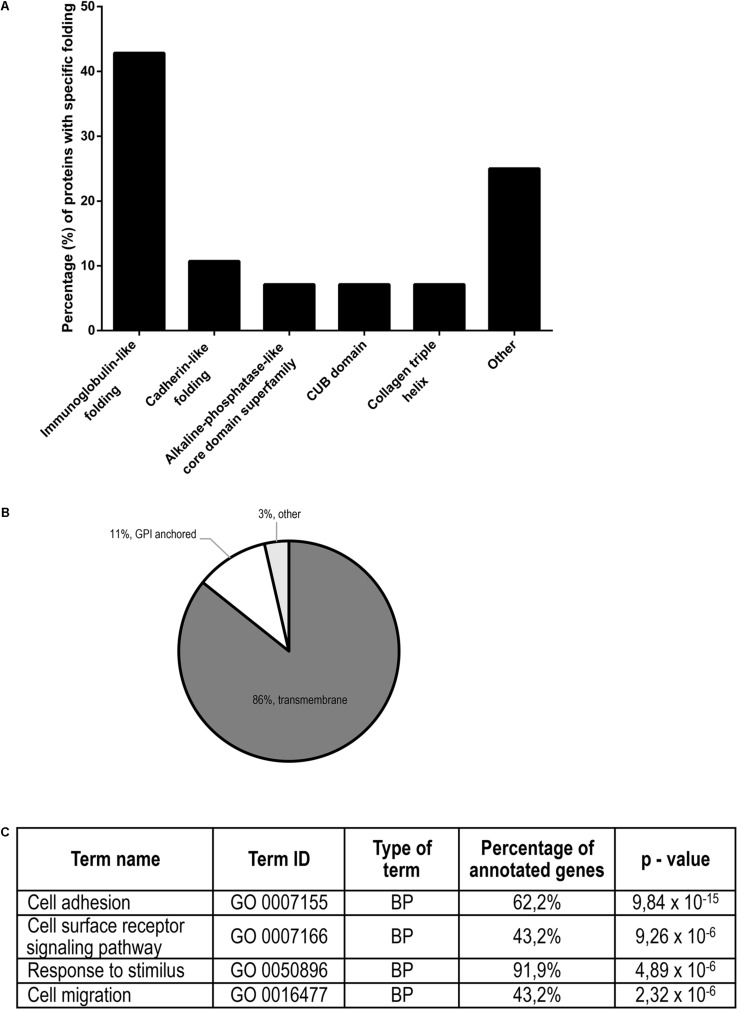
Structural and functional annotation of potential TIGKs cell surface substrates of gingipains. **(A)** Structural similarity (or diversity) of potential gingipain substrates on the cell surface. The majority of the proteins have an immunoglobulin-like fold, followed by CUB domains, cadherin-like fold, collagen triple helix and protein-kinase domain. **(B)** Gingipain substrates were divided according to their attachment to the membrane: transmembrane, GPI-anchor or other types of membrane binding. **(C)** Functional annotation of all gingipain substrates determined by Gene Ontology. Most highly enriched biological processes (BP) with appurtenant portion of the annotated genes are ranked by decreasing *p*-value.

Proteins identified in conditioned media after treatment with the three gingipains at 4 nM concentration were assessed by protein domain annotation using the InterPro interface. The majority (approximately 45%) of the cleaved proteins have immunoglobulin folds, followed by cadherin, collagen and CUB domains (Figure 6A). Furthermore, we analyzed the cellular location of shedded/degraded proteins, which revealed that a majority of the identified gingipain substrates were transmembrane proteins (86%) (Figure 6B). This suggests that gingipains, even though they have different specificities, cleave a structurally related group of proteins from the cell surface. Finally, we performed functional analyses of gingipain targets using g:Profiler (Reimand et al., 2007, 2016), where gene ontology annotation showed a significant enrichment of proteins involved in cell adhesion, cell migration, and signaling through cell-surface receptors (Figure 6C). The majority of all identified substrates with all three tested gingipains were connected to cellular adhesion, consistent with our microscopic observations (Figure 2).

## Discussion

Periodontitis is a major global health issue with an estimated incidence between 40% (Eke et al., 2016) and 70% (Silva-Boghossian et al., 2009) of adults in developed and developing countries, respectively. It is an inflammatory disease and the most common cause of tooth loss in adults. The application of proteomics to the study of periodontitis has increased in popularity in recent years, mainly because of the rapid development of the technology. While several proteomic studies related to periodontitis have been performed (reviewed in Bostanci and Bao, 2016), this is the first global proteomic study of the extracellular gingipain degradome. Emerging evidence implicates gingipains as sheddases, and many different cell surface proteins can be cleaved by gingipains in the proximity to the cell membrane, including CD46 (Mahtout et al., 2009), Syndecan-1 (Andrian et al., 2006), and TREM-1 (Bostanci et al., 2013). However, only a few proteins were shown to be shed from the epithelial cells by gingipains, and they all belong to different protein groups with different functions. Therefore, we aimed to perform a global proteomic analysis of all potential TIGK-cell-membrane targets of gingipains in order to better understand the complex interface between *P. gingivalis* and host epithelial barriers.

Infection of TIGK cells with *P. gingivalis* resulted in substantial protein degradation at high MOI, which may reflect the situation in advanced periodontitis, where numerous bacteria are associated with inflamed tissue. Moreover, wild-type *P. gingivalis* (W83) was by far the most efficient at degrading the proteins, consistent with the major role of gingipains (Figure 1A). Furthermore, *ΔrgpAB*, expressing only Kgp was found to be the least efficient, in keeping with previous reports showing lower general proteolytic activity of Kgp in comparison to Rgps (Tada et al., 2003). This suggests that R-gingipains play a major role in degrading cell surface proteins. However, gingipains have a dual role in periodontitis, in addition to their proteolytic function, they also contain HA domains which facilitate adhesion of *P. gingivalis* to epithelial cells (Tokuda et al., 1996). HA domains were also found to have an important role in proteolysis of membrane proteins, as inhibition of gingipains with KYT did not prevent *P. gingivalis* wild type (W83) from degrading more cell-surface proteins compared to the gingipain-null strain (Δ*kgp*Δ*rgpAB*) (Figure 1B). This suggests that the likely role of adhesion domains in *P. gingivalis*-mediated cell surface proteolysis is to enable the bacteria to adhere to epithelial cells, thus bringing the proteases in the proximity of membrane proteins (Chen et al., 2001; Chen and Duncan, 2004; Pathirana et al., 2006). Thus, the overall conclusion is that strains with gingipain-adhesion domain complexes are more potent at degrading host membrane proteins. However, even at low MOI, degradation was still observed, regardless of the activity or the presence of gingipains, suggesting that they are not the only proteases responsible for proteolysis. This is supported by the findings that while gingipains represent up to 85% of overall proteolytic activity of *P. gingivalis*, these bacteria also secrete other proteases, such as PrtT protease, Tpr protease, Lys-peptidase, oligopeptidase and an array of di- and tri-peptidyl aminopeptidases, which apparently also contribute to degradation of cell surface proteins (Potempa et al., 1997; Grenier and La, 2010).

To discriminate between the role of gingipains and other *P. gingivalis* effects in TIGK cell surface proteolysis, we performed a global proteomic analysis with purified gingipains. In total, 28 membrane proteins were identified as gingipain substrates with majority being cleaved by RgpB, followed by HRgpA and Kgp (Figure 3). RgpB and HRgpA cleaved many common proteins and generated many identical peptides (Figure 4) as they have almost identical catalytic domain and the same proteolytic specificity (Potempa et al., 1998; Ally et al., 2003). On the other hand, Kgp was least efficient with only eight identified membrane substrates and targeted few different proteins, most likely due to its different specificity (de Diego et al., 2014). The majority of identified proteins were connected to cellular adhesion, followed by cell migration and signaling using cell-surface receptors. This is consistent with our observations that gingipains mainly affect epithelial cell adhesion (Figure 2), suggesting that gingipain-mediated shedding and degradation play important roles in disrupting cell–cell contacts and cell-ECM adhesion. Furthermore, gingipains could dysregulate important signaling pathways by receptor degradation, possibly having a role in periodontal bacterial invasion beyond simple degradation of ECM components and alteration of cell adhesion. One such substrate is TRFC, which was identified as a target of gingipains. Using Kgp, relatively less potent in comparison with RgpB and HRgpA, we were able to demonstrate that gingipains can actually first shed their targets from the cell surface and then immediately degrade them. This sequence of events will be more pronounced in the physiological milieu with a plethora of secreted bacterial proteases. Another interesting target is CD44 due to its potential role in *P. gingivalis*-induced anoikis of epithelial cells, a type of programmed cell death initiated by the lack of cell contacts in the case of adhesive cells (Chiarugi and Giannoni, 2008). CD44 is the receptor for hyaluronic acid, a vital component of ECM and crucially important for cell–cell and cell-ECM signaling. CD44 plays a significant role in anoikis induction (Bunek et al., 2010; Cieply et al., 2015); therefore, the observed degradation of CD44 on gingival keratinocytes by gingipains could be one of the inducers of anoikis leading to a damage of the gingival epithelium during progression of periodontitis. Anoikis has been observed in bacterial infections and was connected to the effect of bacterial proteases on host cells (Beaufort et al., 2013; DuMont and Cianciotto, 2017).

On the basis of the results herein we propose the following mechanism for gingipain-mediated proteolysis of epithelial cell membrane proteins. Initially, gingipains, and possibly other proteases, shed their targets from cell surface, which is quickly followed by degradation of the cleaved ectodomain(s), in accord with an earlier hypothesis (Hocevar et al., 2018), This is consistent with the fact that shedding might cause the cleaved proteins to unfold, possibly exposing other cleavage sites and increasing the propensity for degradation. Similar results were obtained in numerous other studies, where shedding/cleavage of membrane protein was observed at lower gingipain or *P. gingivalis* concentrations or shorter incubation times. Further degradation of large fragments occurred when the incubation time was prolonged or gingipain concentration increased (Sugawara et al., 2000; Oleksy et al., 2002; Tada et al., 2003; Mezyk-Kopec et al., 2005; Yun et al., 2007; Bostanci et al., 2013; Wilensky et al., 2014). The difference in the extent of degradation by gingipains at lower and higher concentration can be also viewed as the difference between the early and advanced stage of periodontitis. At the beginning of the disease, bacteria must adhere to the oral tissues and survive in that microenvironment. They need nutrients, which can be provided by gingipain-mediated degradation of host proteins, and they must reach equilibrium with the host immune system in order to avoid killing and removal. However, when the disease develops, the gingipain action shifts from pro-survival to pro-invasive and becomes destructive. In this case the immune system no longer protects the host cells from the bacteria, and additionally bacterial growth and virulence is enhanced by inflammatory degradation products (reviewed in Kadowaki et al., 2000; Potempa et al., 2000; Popadiak et al., 2007; Guo et al., 2010; Hajishengallis, 2010; Hajishengallis et al., 2011, 2013; Damgaard et al., 2015).

In summary, this study provides novel insights into gingipains effect on gingival keratinocytes. We have shown that gingipains and their adhesion domains are vital for *P. gingivalis* proteolytic attack on cell surface proteins of the oral epithelium. Our results suggest that shedding is only the first step in the degradation process of cytoplasmic membrane proteins by gingipains, especially at lower concentrations and shorter incubation times, a reflection of the early phase of periodontitis development. As the concentration of gingipains rises, the degradation of extracellular keratinocyte surface proteins by gingipains starts to dominate over specific proteolytic cleavages, which is in accordance with extensive damage to the gingival epithelium at advanced, severe stages of periodontitis. Among proteins identified as substrates for gingipains, the majority was associated with cell adhesion, which indicated that gingipains may induce anoikis due to disruption of cell–cell and cell-ECM contacts. We identified CD44, one of the most important adhesion receptors on keratinocytes, as a prominent target for gingipains. Degradation of CD44 may induce anoikis, but this hypothesis needs to be investigated in the future. The majority of the other proteins identified as putative substrates of gingipains were observed as such for the first time, opening a fertile ground for future research of mechanisms by which invading bacteria impact different tissues components in the oral cavity.

## Data Availability Statement

The datasets generated for this study can be found in the in the ProteomeXchange database with identifier PXD015679.

## Author Contributions

KH, AW, and MV carried out the experiments. KH, MV, MF, JK, JP, and BT analyzed the data. KH, MV, BT, and JP wrote the manuscript, while all the other authors commented on it. BT and JP conceived the project and supervised it with the help of JK. RL and BP prepared TIGK cells and isolated and purified gingipains, respectively.

## Conflict of Interest

The authors declare that the research was conducted in the absence of any commercial or financial relationships that could be construed as a potential conflict of interest.
