# Supplementary material for: Proteolysis of Gingival Keratinocyte Cell Surface Proteins by Gingipains Secreted From Porphyromonas gingivalis – Proteomic Insights Into Mechanisms Behind Tissue Damage in the Diseased Gingiva
Source: Front Microbiol. 2020 Apr 28;11:722. doi: 10.3389/fmicb.2020.00722 (PMC7198712; doi:10.3389/fmicb.2020.00722)
Supplement: Supplementary file 1 [file Data_Sheet_1.docx]

Supplementary Material

# Supplementary Figures


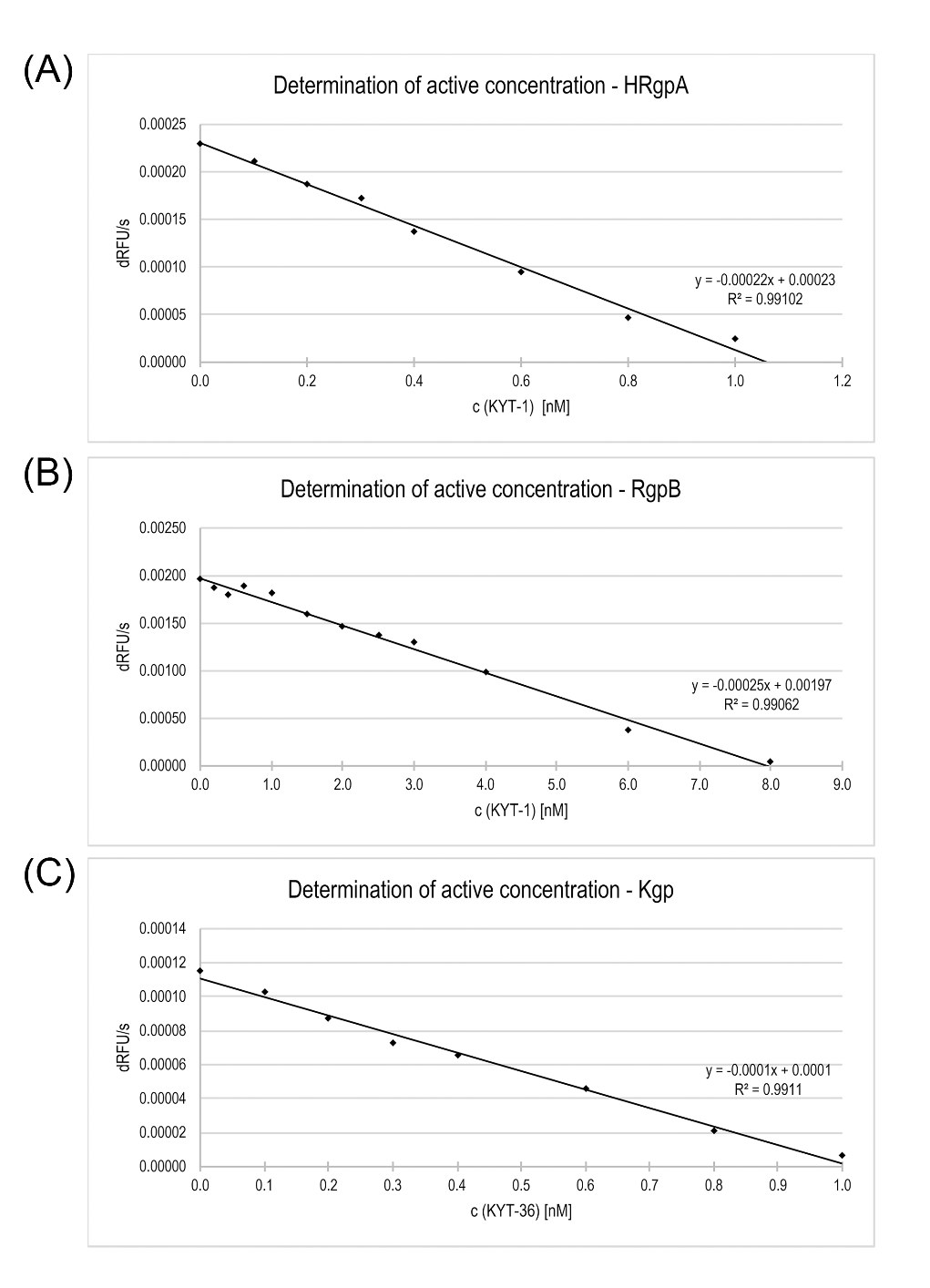


**Supplementary Figure 1: Determination of active concentration of gingipains**

Gingipains (10 nM final protein concentration) were titrated with different concentrations of their respective inhibitors in transparent 96-well plates. Absorbance of chromogenic substrates was measured at 405 nm. Active concentration was determined using linear regression analysis. **A**) Active concentration determination for HRgpA. **B**) Active concentration determination for RgpB. **C**) Active concentration determination for Kgp.


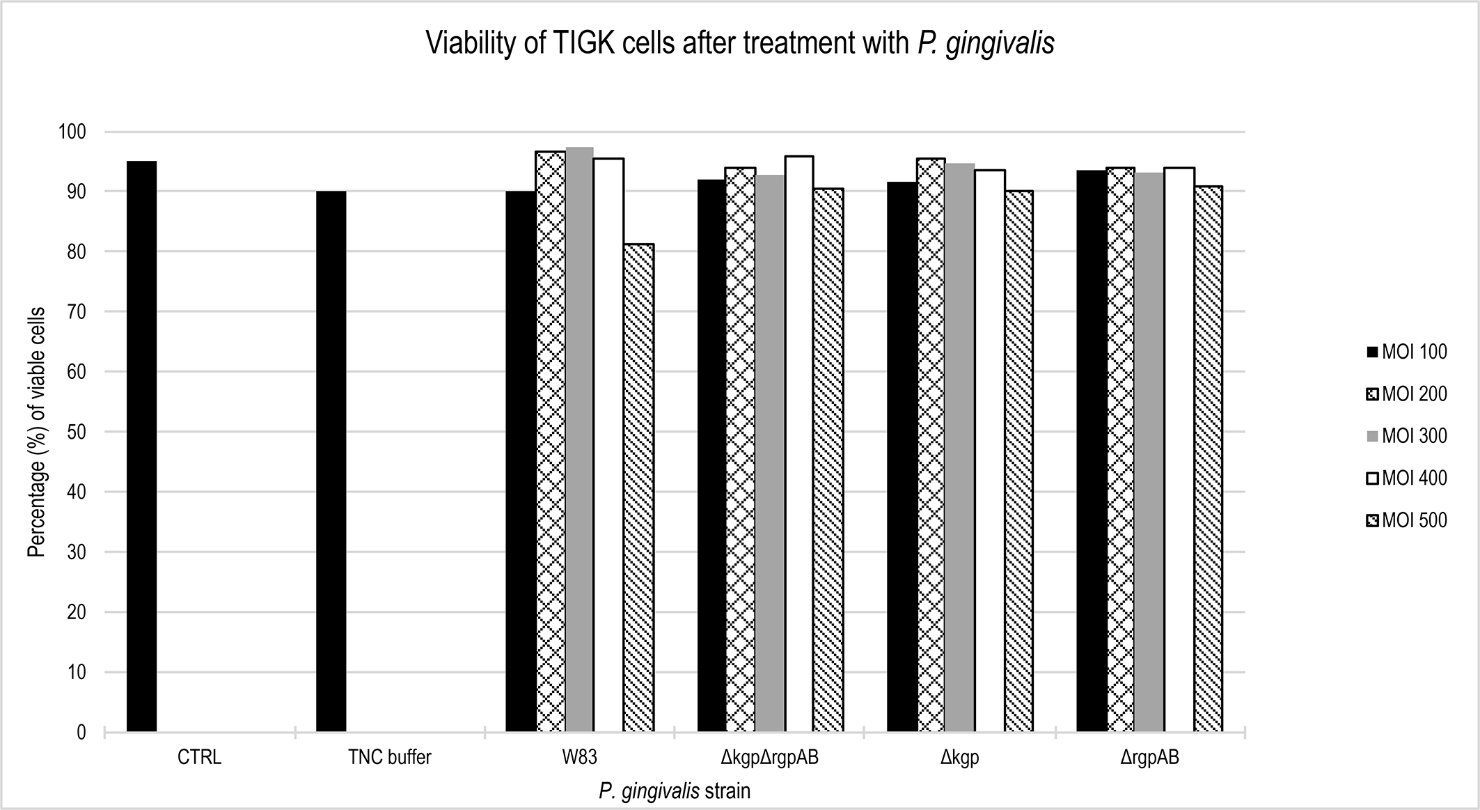


**Supplementary Figure 2:** **Viability of TIGKs after treatment with *P. gingivalis*.**

Intact keratinocytes were treated for 45 min with four different strains of *P. gingivalis* in order to test their viability. Standard Annexin V-FITC and propidium iodide staining and flow cytometry was applied to show that the cells used in the experiments were viable.


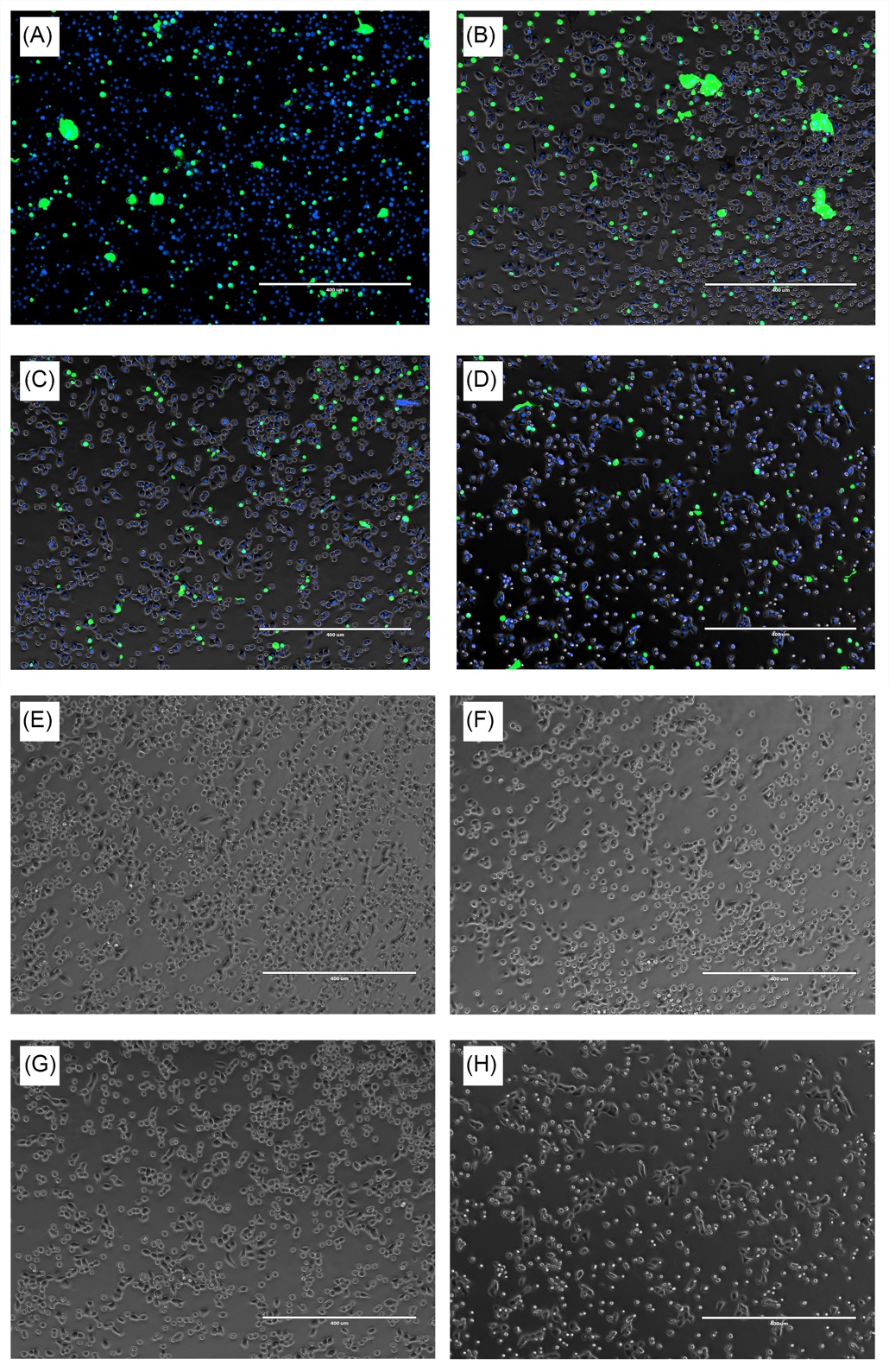


**Supplementary Figure 3:** **Viability of TIGK cells after 45-minute treatment with** **4** **nM gingipains.**

Intact TIGKs were treated with TNC buffer as a control or with gingipains for 45 minute with (A) and (E) TNC buffer, (B) and (F) 4 nM RgpB, (C) and (G) 4 nM HRgpA and (D) and (H) 4 nM Kgp. Viability of the cells was determines using using ReadyProbes Cell Viability Imaging Kit, Thermofisher Scientific. Live cells are stained in blue, while those with compromised plasma membrane integrity in green for photos A – D, while brightfield was applied for photos E – H.

**
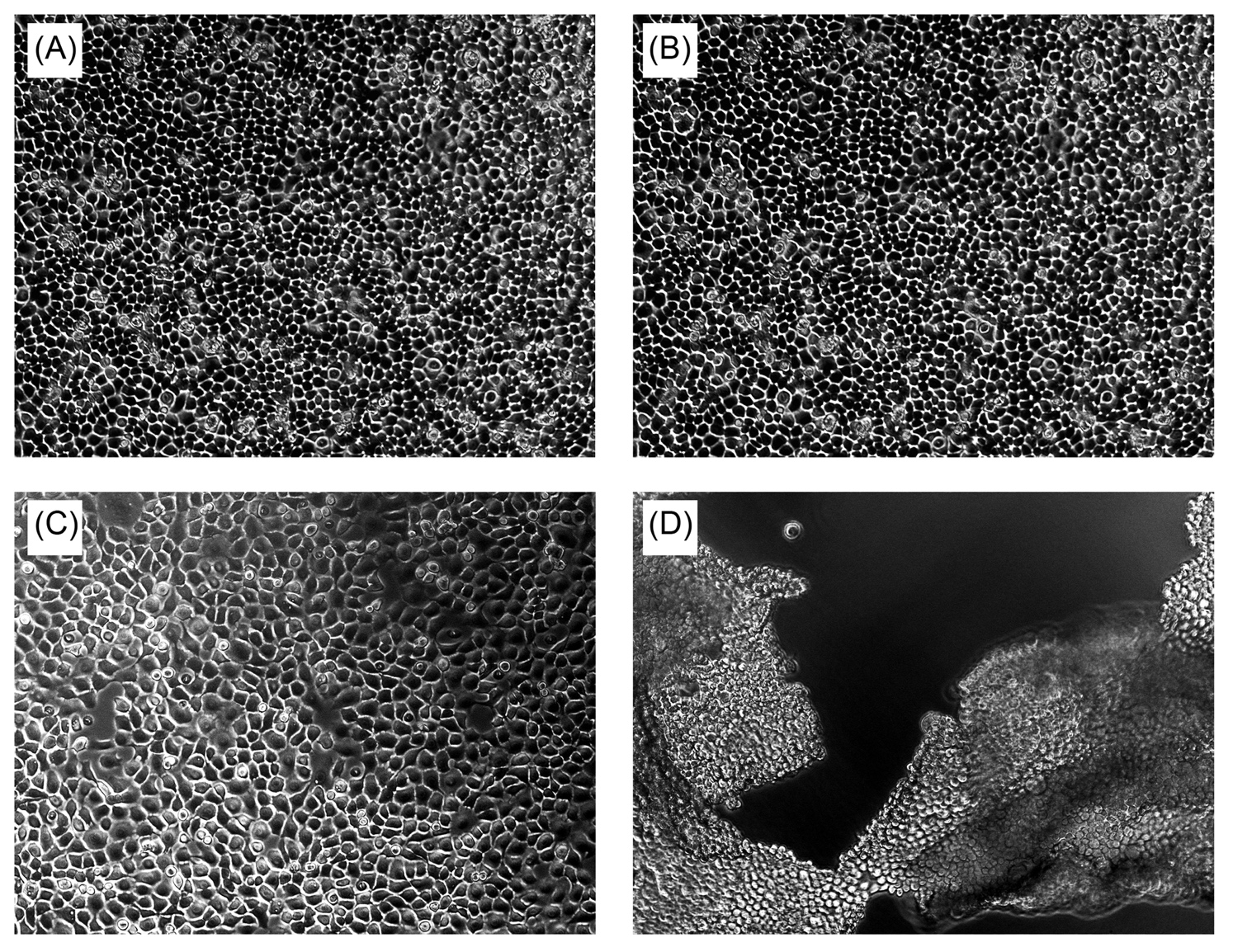
**

**Supplementary Figure 4: The effect of 100 nM concentration of Kgp on TIGKs after 30-minute treatment.**

Intact TIGKs were treated with TNC buffer as a control or with Kgp in different concentrations to compare the effect of bacterial proteases on TIGKs. The pictures were taken using light microscopy.
**A**) Cell morphology at the beginning of the treatment (t = 0 min); **B**) after 30 minutes of treatment with TNC buffer only; **C**) after 30 minutes of 4 nM Kgp treatment; and **D**) after 30 minutes of 100 nM Kgp treatment.

**Supplementary Figure 5: Mass spectrometric identification of TIGK-cell proteins in conditioned media with gingipains at 75 nM concentration*.***

**(A)** Distribution of SCR and cell localization of proteins in the samples identified with mass spectrometry. Intact TIGK cells were treated with purified gingipains RgpB, RgpA and Kgp at 75 nM concentration. The pie charts for the RgpB, RgpA and Kgp treatment represent the ratios between the proteins with decreased abundance (SCR ≤ 0.33), unchanged abundance (0.33 < SCR < 3) and increased abundance (SCR ≥ 3) after gingipain treatment. Proteins with SCR ≥ 3 were further divided into 4 categories, depending on their cellular localization (membrane, cytoplasm, nucleus and other locations). The higher percentages of proteins in the group with SCR ≤ 0.33 is likely to be the consequence of more extensive degradation by gingipains. **(B)** List of potential gingipain targets with indication in which sheddome (RgpB, HRgpA and Kgp) proteins were detected. Only proteins localized on the cell membrane, identified with minimum 2 peptides and with minimum three-fold increase in SCR were considered.

# Original blots and gels


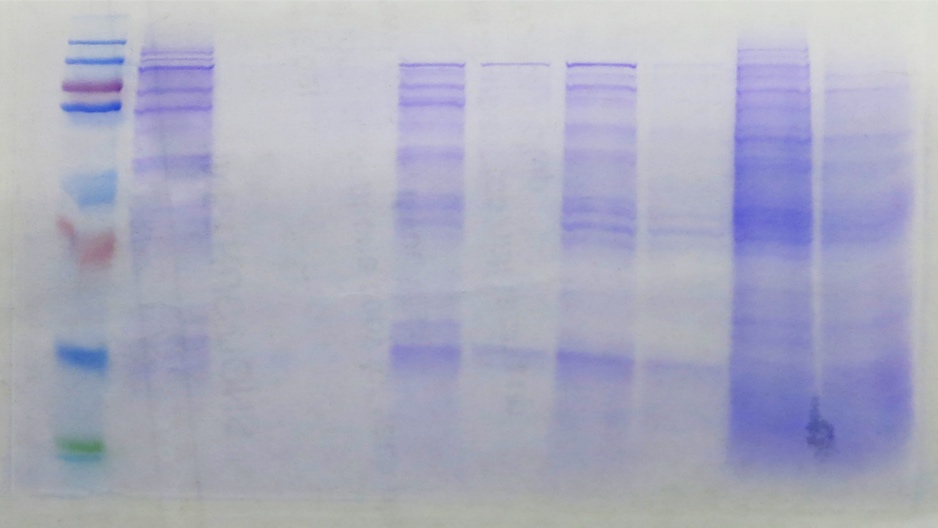


Figure 1A, original gel


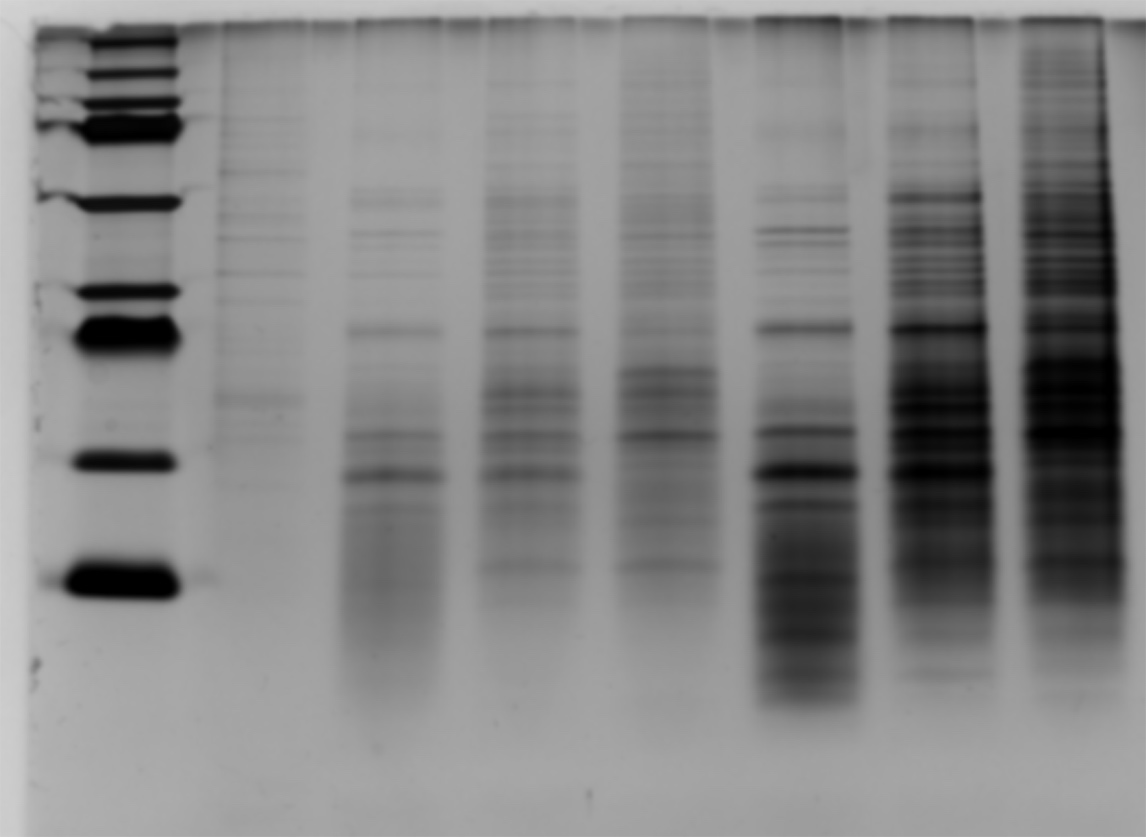


Figure 1B, original gel


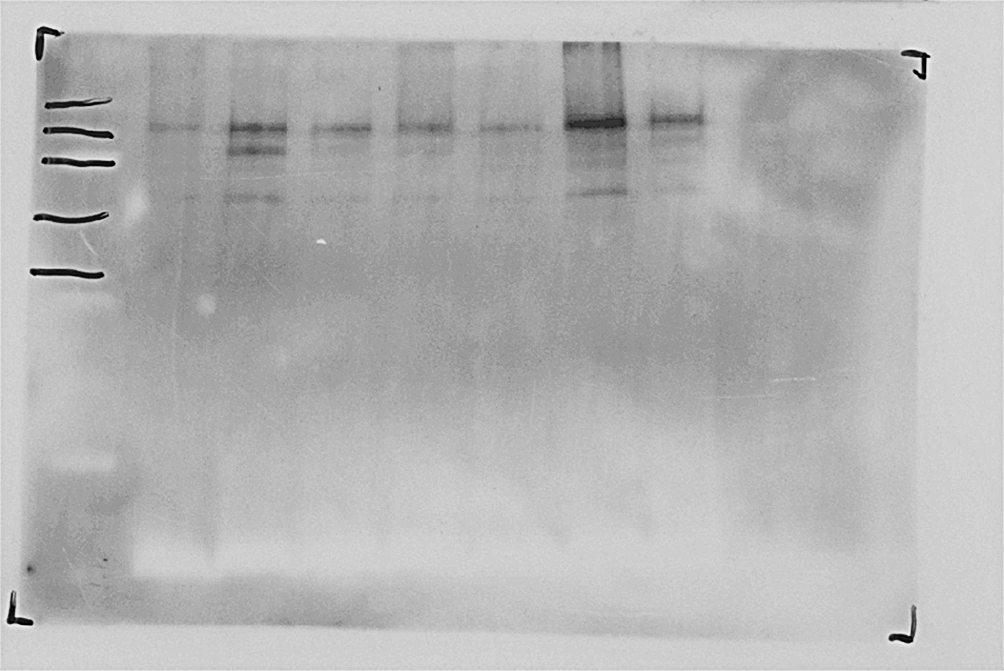


Figure 5A, original blot


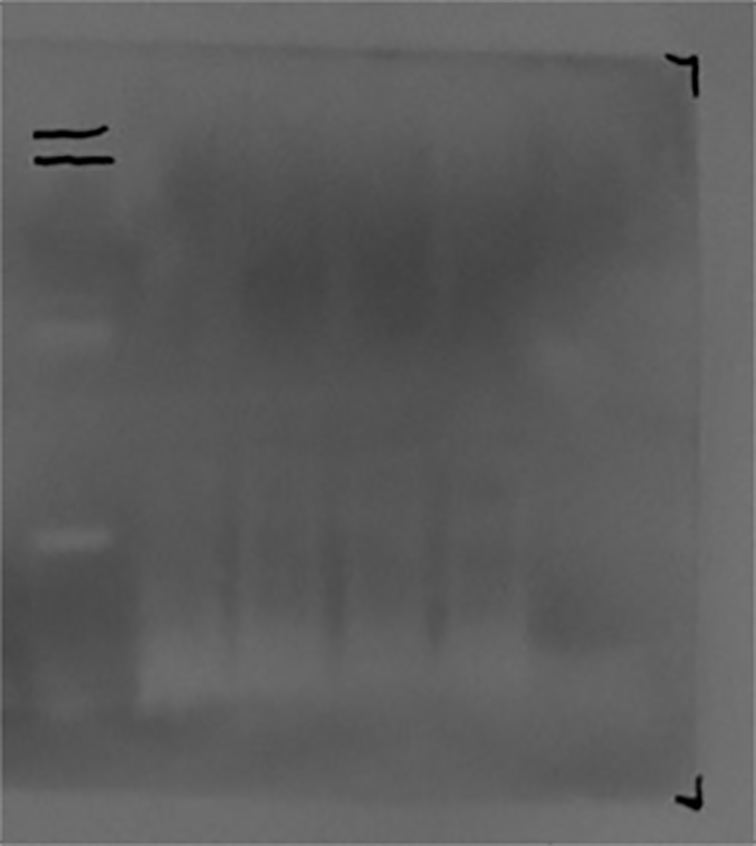


Figure 5B, original blot


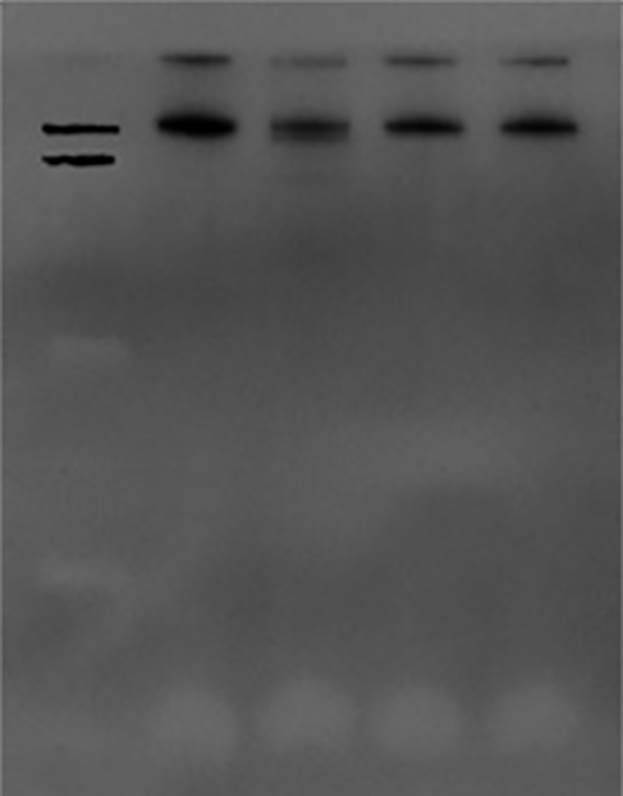


Figure 5C, original blot
